# E/e' ratio combined with left ventricular mass index predicts HFpEF rehospitalization risk in stage 3–4 chronic kidney disease patients: a retrospective cohort study

**DOI:** 10.3389/fcvm.2026.1758192

**Published:** 2026-05-28

**Authors:** Manqiong Xie, Yunchong Chen, Dan Ge, Ting Jiang

**Affiliations:** 1 Department of Ultrasound, Hangzhou Hospital of Traditional Chinese Medicine (Hangzhou TCM Hospital Affiliated to Zhejiang Chinese Medical University), Hangzhou, Zhejiang, China; 2 Department of Ultrasound, Sir Run Run Shaw Hospital Zhejiang University School of Medicine, Handzhou, Zhejiang, China

**Keywords:** chronic kidney disease, e/e′ ratio, HFPEF, left ventricular mass index, rehospitalization

## Abstract

**Introduction:**

Patients with heart failure with preserved ejection fraction (HFpEF) and coexisting chronic kidney disease (CKD) have poor outcomes. This study assessed whether a combined echocardiographic indicator could improve risk stratification for this high-risk group.

**Methods:**

In a retrospective study of 254 adults with HFpEF and stage 3–4 CKD, we evaluated an indicator combining elevated E/e' ratio (≥15) and left ventricular hypertrophy (LVH). The primary outcome was rehospitalization for HFpEF. Statistical analyses included Cox regression, C-index, and decision curve analysis.

**Results:**

Over a 2.1-year median follow-up, 26.8% of patients were rehospitalized. The combined indicator was a strong, independent predictor of this outcome. It demonstrated superior discrimination (C-index 0.74) compared to either E/e' (0.67) or LVH (0.65) alone, showing clinical utility across a range of risk thresholds.

**Conclusion:**

A combined echocardiographic indicator integrating E/e' and LVMI improves risk stratification in patients with HFpEF and advanced CKD.

## 1 Introduction

Heart failure with preserved ejection fraction (HFpEF) represents a growing global health challenge, accounting for over half of all heart failure cases. It is characterized by significant morbidity, high rates of hospitalization, and poor survival (1). The clinical landscape of HFpEF is further complicated by a high prevalence of comorbidities, among which chronic kidney disease (CKD) is particularly prominent and impactful. Epidemiological studies indicate that CKD coexists in 40% to 60% of patients with HFpEF, establishing a strong bidirectional relationship where the presence of one condition accelerates the progression of the other (2, 3). This interplay, often termed cardiorenal syndrome, involves shared pathophysiological mechanisms, including systemic inflammation, neurohormonal activation, and hemodynamic overload, which collectively contribute to adverse clinical outcomes (4). Consequently, patients with both HFpEF and moderate-to-severe CKD (stages 3–4) face a substantially elevated risk of mortality and recurrent hospitalization compared to those with either condition alone (5).

Accurate risk stratification is critical for guiding therapeutic decisions and improving outcomes in this high-risk population. Echocardiography remains the cornerstone for the non-invasive evaluation of cardiac structure and function in HFpEF. The E/e' ratio, which is the ratio of early mitral inflow velocity (E) to early diastolic mitral annular velocity (e'), is a well-established marker of left ventricular (LV) filling pressures and diastolic dysfunction, serving as a key diagnostic and prognostic parameter in HFpEF (6). Concurrently, the left ventricular mass index (LVMI) provides a quantitative measure of cardiac remodeling and hypertrophy, a common pathological finding in HFpEF that is strongly associated with adverse cardiovascular events (7). Although both E/e' and LVMI are individually recognized for their prognostic value, their utility can be influenced by the complex physiological changes inherent to advanced CKD, such as volume overload and altered hemodynamics.

Despite the individual prognostic significance of E/e' and LVMI, there is a lack of evidence regarding the predictive power of a combined indicator incorporating both metrics for risk stratification in patients with concomitant HFpEF and stage 3–4 CKD. Given that E/e' reflects hemodynamic status (LV filling pressure) and LVMI represents structural remodeling (hypertrophy), a composite measure could offer a more holistic and robust assessment of pathophysiology, potentially improving the identification of patients at the highest risk for adverse events. Such a tool could enhance clinical decision-making, optimize follow-up strategies, and aid in the selection of patients who may benefit most from targeted therapies like SGLT2 inhibitors, which have shown promise in this cardiorenal population (8).

Therefore, this retrospective cohort study aimed to investigate the hypothesis that a combined indicator of the E/e’ ratio and LVMI could more effectively predict the risk of rehospitalization for heart failure in patients with HFpEF and stage 3–4 CKD. By evaluating the combined predictive utility of these accessible echocardiographic parameters, we sought to develop a simple, non-invasive tool to improve risk stratification in this challenging and growing patient population.

## 2 Materials and methods

### 2.1 Study design

This single-center retrospective cohort study utilized electronic medical records (EMR) to assess the predictive value of combining the E/e′ ratio and LVMI for HFpEF rehospitalization in patients with stage 3–4 CKD. Data were collected from January 1, 2018, to December 31, 2023, ensuring a minimum follow-up period of one year. The study protocol was approved by the Institutional Review Board of Hangzhou Hospital of Traditional Chinese Medicine (No: 2024LL003). Informed consent was waived due to the retrospective nature of the study, which involved anonymized data analysis without any intervention, in compliance with local regulations and the Declaration of Helsinki. All patient data were handled confidentially following strict privacy protection protocols. The study was conducted in accordance with the STROBE guidelines for reporting observational studies.

### 2.2 Study population

The study cohort was identified through a query of the hospital's electronic medical record system using International Classification of Diseases, Tenth Revision (ICD-10) codes for chronic kidney disease (N18.3, N18.4) and heart failure (I50.3, I50.8, I50.9). All patients aged 18 years or older with at least one inpatient or two outpatient encounters between January 1, 2018, and December 31, 2023, were initially screened. Patient selection followed a multi-stage process: (1) automated identification based on diagnostic codes and laboratory-confirmed CKD stage; (2) detailed manual review of medical records by two independent physicians to verify the diagnosis of HFpEF and confirm eligibility against all inclusion and exclusion criteria using standardized data extraction forms; and (3) final adjudication by a heart-kidney committee in cases of disagreement or uncertainty.

Inclusion criteria comprised all of the following: (1) Age ≥18 years; (2) Chronic kidney disease, stages 3–4, defined by an estimated glomerular filtration rate (eGFR) between 15 and 59 mL/min/1.73 m^2^, calculated using the CKD-EPI 2009 equation, on at least two occasions 90 days apart, consistent with KDIGO guidelines (9); (3) First-time diagnosis of heart failure with preserved ejection fraction (HFpEF), fulfilling the 2016 ESC guidelines criteria (10), which require the presence of signs and/or symptoms of heart failure, LVEF ≥50%, and elevated levels of natriuretic peptides (BNP ≥35 pg/mL or NT-proBNP ≥125 pg/mL) accompanied by relevant structural heart disease (left ventricular hypertrophy/left atrial enlargement) or diastolic dysfunction on echocardiography; (4) Availability of a comprehensive echocardiographic examination including pulsed-wave Doppler and tissue Doppler imaging performed within 3 months of the index HFpEF diagnosis.

Exclusion criteria encompassed any of the following: (1) Significant primary valvular heart disease (moderate or severe stenosis/regurgitation) or congenital heart disease; (2) History of heart transplantation or left ventricular assist device implantation.; (3) End-stage malignant disease (e.g., metastatic cancer) or other life-limiting conditions with an expected survival of less than 6 months; (4) Hemodynamically significant arrhythmias at the time of the index echocardiogram that preclude accurate diastolic parameter measurement; (5) Incomplete medical records or key variable data missing exceeding 20% (*n* = 14); (6) Loss to follow-up (i.e., no documented clinical encounter) within 6 months after the index diagnosis unless a primary endpoint (HFpEF rehospitalization) occurred earlier (*n* = 12). Patients lost to follow-up were excluded from the primary analysis but were included in a sensitivity analysis to assess the potential impact of informative censoring.

In a prespecified sensitivity analysis to test the robustness of the results, the HFpEF patient cohort was redefined using updated diagnostic criteria based on the 2021 ESC Guidelines for the diagnosis and treatment of acute and chronic heart failure and the Universal Definition and Classification of Heart Failure (11, 12). Specifically, patients included in this analysis were required to meet all of the following criteria: (1) presence of signs and/or symptoms of heart failure; (2) left ventricular ejection fraction (LVEF) ≥ 50%; and (3) objective evidence of cardiac structural and/or functional abnormalities, including left ventricular hypertrophy (LVMI >115 g/m^2^ in men or >95 g/m^2^ in women) or left atrial enlargement (left atrial volume index >34 mL/m^2^), elevated natriuretic peptide levels (BNP ≥35 pg/mL or NT-proBNP ≥125 pg/mL), or echocardiographic evidence of diastolic dysfunction (average E/e' ratio >14 or septal E/e' > 15) (3–6).

The sample size was estimated *a priori*. Based on the rule of thumb requiring at least 10 events per variable (EPV) for Cox regression models and anticipating a primary endpoint (HFpEF rehospitalization) incidence of approximately 10% in this population, a minimum of 200 patients was deemed necessary to ensure robust statistical power for the planned multivariable analyses adjusting for key covariates.

### 2.3 Variable definitions and data collection

The primary exposure variables were echocardiographic parameters defining cardiac diastolic function and structure. Left ventricular filling pressure was assessed by the E/e' ratio, defined as the ratio of early mitral inflow velocity (E) to the early diastolic mitral annular velocity (e') obtained from the interventricular septum using tissue Doppler imaging. An elevated E/e' ratio was defined as ≥15, a well-validated cutoff indicating significantly increased left ventricular filling pressure, based on recommendations from the American Society of Echocardiography and the European Association of Cardiovascular Imaging (6). Left ventricular mass index (LVMI, g/m^2^) was calculated from two-dimensional linear measurements according to the 2015 ASE/EACVI guidelines for chamber quantification (13), with left ventricular hypertrophy defined as an LVMI ≥115 g/m^2^ for men and ≥95 g/m^2^ for women. To enhance practical applicability, we derived a single binary combined parameter for risk stratification. This combined indicator was defined as positive (score of 1) only if both an elevated E/e' ratio (≥15) and the presence of LVH (LVMI ≥115 g/m^2^ for men and ≥95 g/m^2^ for women) were present, and negative (score of 0) otherwise. This approach integrates functional and structural thresholds into a single, readily interpretable clinical tool. This combination integrates a key functional abnormality (diastolic dysfunction) with a structural alteration (ventricular hypertrophy), providing a more comprehensive pathophysiological basis for risk stratification in HFpEF, as supported by contemporary literature (14). The primary outcome was the first rehospitalization for HFpEF following the index diagnosis. This endpoint was rigorously adjudicated using standardized criteria (15, 16), requiring an unplanned hospitalization lasting ≥24 h due to worsening signs and symptoms of heart failure, supported by objective evidence such as elevated NT-proBNP levels (>125 pg/mL), radiographic evidence of pulmonary congestion, or the need for intravenous decongestive therapy, with heart failure documented as the primary discharge diagnosis and alternative primary causes (e.g., pneumonia) excluded. Covariates were selected *a priori* based on their established clinical relevance to HFpEF prognosis and potential for confounding. These included demographic factors (age, sex, body mass index), clinical parameters (systolic blood pressure at baseline, New York Heart Association functional class, estimated glomerular filtration rate, hemoglobin level), key comorbidities (history of diabetes mellitus, coronary artery disease, atrial fibrillation), and background medical therapies known to influence heart failure outcomes (use of renin-angiotensin-aldosterone system inhibitors and beta-blockers). To account for overall comorbidity burden, the Charlson Comorbidity Index (17) was also calculated for each patient.

### 2.4 Data quality control

All transthoracic echocardiographic examinations were performed by experienced sonographers using commercially available ultrasound systems (Vivid E95 or Vivid S70, GE Healthcare, Chicago, IL, USA) equipped with M5S or M6S phased-array transducers. Standard two-dimensional, spectral Doppler, and tissue Doppler imaging (TDI) were acquired following the American Society of Echocardiography guidelines (11). Digital clips of three consecutive cardiac cycles were stored in cine-loop format for offline analysis. To ensure measurement accuracy and minimize observer bias, the offline analysis of the key exposure variables (E/e’ ratio and LVMI) was performed using a dedicated workstation running EchoPAC software (version 203, GE Healthcare). This analysis was conducted by two independent cardiologists with Level III echocardiography certification, who were blinded to all clinical data, laboratory results, and patient outcomes. The inter-observer reliability for the continuous measurements of E/e' and LVMI was assessed using the intraclass correlation coefficient (ICC) in a random sample of 30 patients. The ICC values demonstrated excellent agreement (ICC for E/e' = 0.94, 95% CI: 0.88–0.97; ICC for LVMI = 0.92, 95% CI: 0.84–0.96). Any discrepancies exceeding 10% between the two readers were resolved by a joint re-review and consensus. The ascertainment of the primary outcome (HFpEF rehospitalization) was performed by two independent physicians via detailed review of hospitalization records, including physician notes, laboratory results, and imaging reports. In cases of disagreement or diagnostic uncertainty, the final adjudication was made by a dedicated heart-kidney committee, whose members were blinded to the patients’ exposure variable status, to avoid diagnostic bias.

### 2.5 Statistical analysis

All analyses were conducted using SPSS version 26.0 and R version 4.5.0 (utilizing packages survival, cmprsk, and mice), with a two-sided *p*-value < 0.05 considered statistically significant. The association between the continuous variables E/e' and LVMI was assessed using Spearman's rank correlation due to their non-normal distribution, visualized with a scatter plot incorporating a locally weighted scatterplot smoothing (LOESS) curve. Baseline characteristics were compared between groups defined by the combined indicator status; categorical variables were presented as frequencies (percentages) and compared using the Chi-square or Fisher's exact test, while continuous variables were expressed as mean ± standard deviation or median (interquartile range) and compared using the independent samples *t*-test or Mann–Whitney *U*-test, as appropriate. Time-to-event analysis was conducted using Kaplan–Meier curves, with between-group differences assessed by the log-rank test. The independent association of the combined indicator with the primary outcome was quantified using univariable and multivariable Cox proportional hazards models, presented as hazard ratios (HRs) with 95% confidence intervals (CIs). The multivariable analysis involved sequential adjustment: Model 1 was unadjusted; Model 2 adjusted for demographic and clinical variables (age, sex, BMI, systolic blood pressure, NYHA class, eGFR, hemoglobin); and Model 3 additionally adjusted for comorbidities and medications (diabetes, coronary artery disease, atrial fibrillation, use of RAASi, beta-blockers). The predictive performance of the combined indicator was evaluated against the individual components by assessing discrimination and reclassification. Discrimination was primarily evaluated using time-dependent receiver operating characteristic (ROC) curves at the 1-year follow-up mark with the R package timeROC, and globally assessed using Harrell's C-index. Reclassification improvement was quantified using the continuous net reclassification improvement (NRI) and integrated discrimination improvement (IDI). Furthermore, the clinical utility of the prediction models was assessed using decision curve analysis (DCA), which estimates the standardized net benefit across a range of threshold probabilities to evaluate the potential clinical value of using the model for intervention decisions. Prespecified subgroup analyses were conducted based on age (≤65/>65 years), sex, diabetes status, and eGFR level (30–44/<30 mL/min/1.73 m^2^) by introducing interaction terms into the Cox model, with results visualized using forest plots. To test the robustness of the findings, sensitivity analyses were performed, which included: (1) a competing risk regression model (Fine-Gray subdistribution hazards model) treating death as a competing event; (2) an analysis addressing informative censoring due to loss to follow-up using multiple imputation under the missing-at-random assumption; and (3) a redefinition of HFpEF according to the updated 2021 ESC guidelines for the diagnosis and treatment of heart failure and the universal definition of heart failure. A two-sided *p*-value < 0.05 was considered statistically significant.

## 3 Results

### 3.1 Baseline characteristics

A total of 254 patients with stage 3–4 CKD and a first-time diagnosis of HFpEF were included in the final analysis. Based on the pre-specified combined echocardiographic indicator, 88 patients (34.6%) were classified into the positive group, while 166 patients (65.4%) constituted the negative group. The baseline characteristics are presented in Table 1. Patients in the combined indicator positive group were significantly older and had higher NT-proBNP levels, lower eGFR, a higher prevalence of atrial fibrillation, and a greater comorbidity burden (all *p* < 0.05). In contrast, there were no significant differences between the two groups in terms of sex distribution, body mass index, systolic blood pressure, hemoglobin level, prevalence of diabetes or coronary artery disease, or the use of key heart failure medications (all *p* > 0.05).

**Table 1:** Baseline characteristics stratified by combined E/e’ and LVMI status.

Characteristic	Overall (*N* = 254)	Combined indicator positive (*n* = 88)	Combined indicator negative (*n* = 166)	Statistical test	*P*-value
Demographics					
Age, years, mean ± SD	70.1 ± 9.8	73.2 ± 8.9	68.5 ± 9.9	*t*-test	<0.001
Female, *n* (%)	137 (53.9)	49 (55.7)	88 (53.0)	Chi-square	0.68
BMI, kg/m², mean ± SD	28.6 ± 5.0	28.9 ± 5.2	28.4 ± 4.9	*t*-test	0.45
Clinical Parameters					
SBP, mmHg, mean ± SD	135 ± 15	136 ± 16	134 ± 15	*t*-test	0.31
NYHA Class III/IV, *n* (%)	115 (45.3)	48 (54.5)	67 (40.4)	Chi-square	0.03
eGFR, mL/min/1.73 m², mean ± SD	31.5 ± 9.5	28.8 ± 8.7	32.9 ± 9.6	*t*-test	<0.001
Hemoglobin, g/L, mean ± SD	115 ± 15	113 ± 16	116 ± 15	*t*-test	0.13
NT-proBNP, pg/mL, median (IQR)	1,120 (510–2,250)	1,925 (950–3,350)	850 (380–1,720)	Mann–Whitney U	<0.001
Comorbidities					
Diabetes Mellitus, *n* (%)	120 (47.2)	44 (50.0)	76 (45.8)	Chi-square	0.52
Coronary Artery Disease, *n* (%)	98 (38.6)	37 (42.0)	61 (36.7)	Chi-square	0.41
Atrial Fibrillation, *n* (%)	96 (37.8)	44 (50.0)	52 (31.3)	Chi-square	0.003
Charlson Index, median (IQR)	5 (4–6)	6 (5–7)	5 (4–6)	Mann–Whitney U	<0.001
Medications, *n* (%)					
RAAS Inhibitors	182 (71.7)	65 (73.9)	117 (70.5)	Chi-square	0.56
Beta-blockers	172 (67.7)	62 (70.5)	110 (66.3)	Chi-square	0.49
Echocardiographic parameters					
LVEF, %, mean ± SD	58.0 ± 4.6	57.8 ± 4.9	58.1 ± 4.4	*t*-test	0.61
E/e’ ratio, median (IQR)	14.6 (11.9–18.3)	18.9 (16.6–22.3)	12.7 (10.6–14.3)	Mann–Whitney U	<0.001
LVMI, g/m², mean ± SD	109.0 ± 25.5	129.5 ± 19.8	98.5 ± 21.5	*t*-test	<0.001

Data are presented as mean ± standard deviation, median (interquartile range), or number (percentage). Missing data for the included cohort (*N* = 254) were minimal: BMI (*n* = 3), hemoglobin (*n* = 5), and NT-proBNP (*n* = 2). *P*-values in bold indicate statistical significance (*p* < 0.05). BMI, body mass index; SBP, systolic blood pressure; NYHA, New York heart association; eGFR, estimated glomerular filtration rate; IQR, interquartile range; NT-proBNP, N-terminal pro-B-type natriuretic peptide; RAAS, renin-angiotensin-aldosterone system; LVEF, left ventricular ejection fraction; E/e’, ratio of early transmitral flow velocity to early diastolic mitral annular velocity; LVMI, left ventricular mass index.

### 3.2 Distribution and correlation of primary echocardiographic parameters

**Figure 1:**
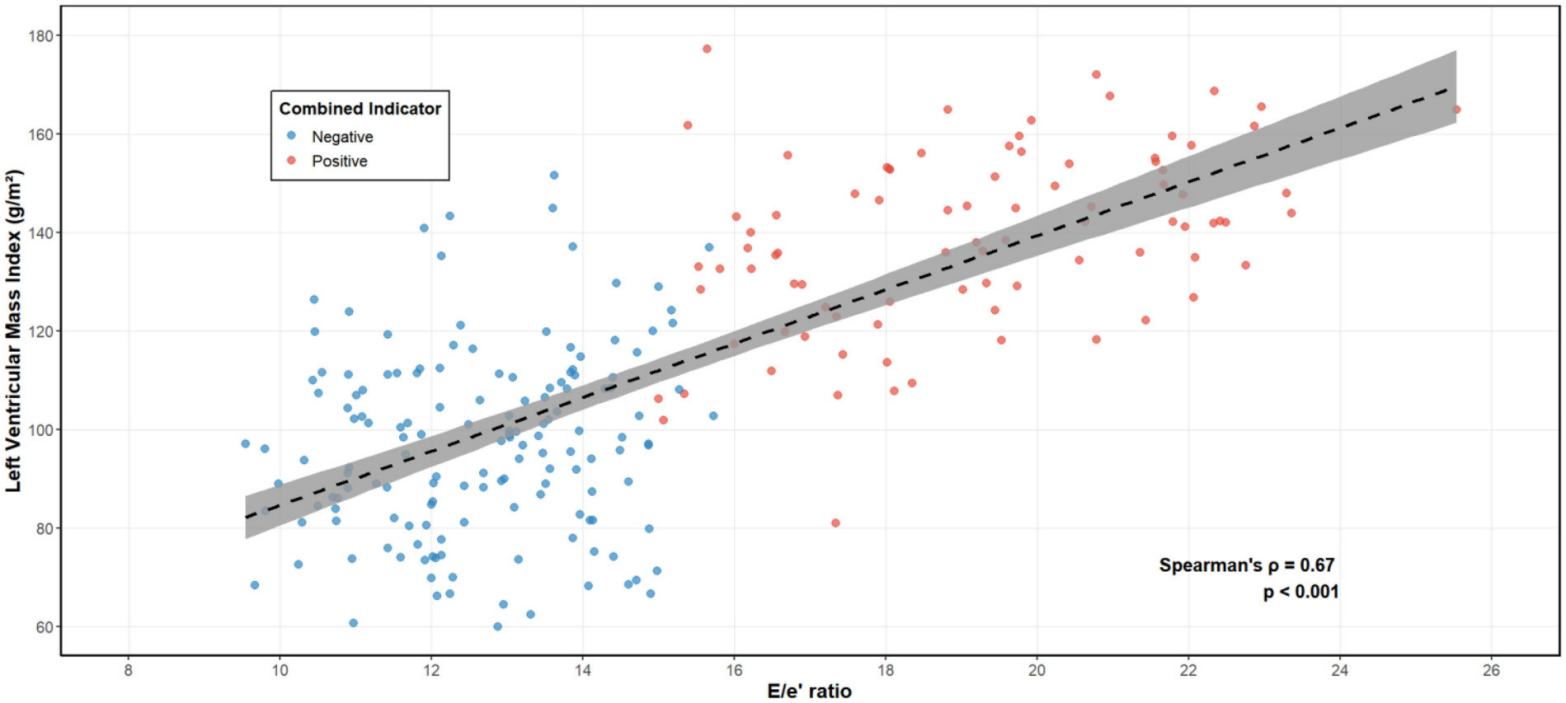
Correlation between E/e’ and LVMI with kernel density distribution. Scatter plot showing the relationship between E/e’ ratio and LVMI in 254 patients with stage 3–4 CKD and HFpEF. Blue circles represent patients with negative combined indicator status (*n* = 166); red circles represent patients with positive combined indicator status (*n* = 88). The black dashed line indicates the linear regression line for the overall cohort. Marginal density plots display the kernel density distributions along each axis. Spearman's correlation coefficient and statistical significance are shown. CKD, chronic kidney disease; E/e’, ratio of early transmitral flow velocity to early diastolic mitral annular velocity; HFpEF, heart failure with preserved ejection fraction; LVMI, left ventricular mass index.

The distribution and correlation between the two primary echocardiographic parameters, E/e' ratio and LVMI, were analyzed in the total cohort. A scatter plot with kernel density distribution illustrated the bivariate relationship between these variables (Figure 1). Spearman's rank correlation analysis revealed a statistically significant, moderate positive correlation between E/e' ratio and LVMI (Spearman's *ρ* = 0.68, *p* < 0.001).

### 3.3 Survival analysis results

**Figure 2:**
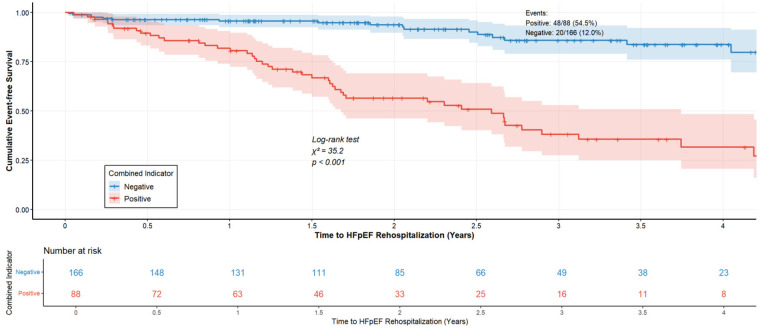
Cumulative HFpEF rehospitalization rate by combined indicator Status. Kaplan–Meier survival curves showing the cumulative incidence of HFpEF rehospitalization stratified by combined echocardiographic indicator status in 254 patients with stage 3–4 chronic kidney disease. The blue line represents patients with negative combined indicator status (*n* = 166), and the red line represents patients with positive combined indicator status (*n* = 88). Shaded areas indicate 95% confidence intervals. The risk table below the plot shows the number of patients at risk at each time point. During a median follow-up of 2.1 years, 68 primary endpoint events occurred (overall incidence rate 26.8%). Statistical comparison performed using log-rank test. HFpEF, heart failure with preserved ejection fraction.

During a median follow-up of 2.1 years (interquartile range: 1.4–3.0 years), 68 primary endpoint events (HFpEF rehospitalization) were recorded, with an overall incidence rate of 26.8%. Kaplan–Meier survival curves demonstrated a significantly higher cumulative incidence of HFpEF rehospitalization in patients with a positive combined indicator compared to those with a negative indicator (log-rank test, *χ*^2^ = 35.2, *p* < 0.001; Figure 2).

The results of the univariable and multivariable Cox proportional hazards regression analyses are presented in Table 2. In the univariable analysis, the presence of the positive combined indicator was strongly associated with an increased risk of HFpEF rehospitalization (HR, 3.52; 95% CI, 2.18–5.67; *p* < 0.001). This association remained significant after sequential adjustment for potential confounders. After adjustment for demographic and clinical variables (Model 2), the HR was 2.89 (95% CI, 1.76–4.74; *p* < 0.001). In the fully adjusted model (Model 3), which included comorbidities and medications, the positive combined indicator remained an independent predictor of the primary outcome (HR, 2.65; 95% CI, 1.59–4.41; *p* < 0.001). The results of the full multivariable Model 3 are shown in Table 2, confirming that besides the combined indicator, higher NYHA class and lower eGFR were also independently associated with increased risk of rehospitalization.

**Table 2:** Univariable and multivariable cox regression analyses for HFpEF rehospitalization.

Variable	Model 1		Model 2*		Model 3^†^	
	HR (95% CI)	*P*-value	HR (95% CI)	*P*-value	HR (95% CI)	*P*-value
Combined indicator						
Positive	3.52 (2.18–5.67)	<0.001	2.89 (1.76–4.74)	<0.001	2.65 (1.59–4.41)	<0.001
Negative (Ref.)	1.00		1.00		1.00	
Age, per 1 year increase	1.02 (0.99–1.05)	0.12	1.03 (1.00–1.06)	0.04	1.03 (1.00–1.06)	0.03
Female Sex	0.98 (0.62–1.56)	0.94	0.99 (0.62–1.59)	0.97	1.01 (0.63–1.63)	0.96
BMI, per 1 kg/m^2^ increase	1.01 (0.97–1.06)	0.56	1.02 (0.97–1.07)	0.43	1.02 (0.97–1.07)	0.39
SBP, per 10 mmHg increase	0.95 (0.79–1.15)	0.61	0.96 (0.79–1.16)	0.66	0.96 (0.79–1.16)	0.67
NYHA Class III/IV	2.15 (1.33–3.48)	0.002	2.01 (1.23–3.28)	0.005	1.89 (1.15–3.10)	0.01
eGFR, per 5 mL/min/1.73 m^2^ decrease	1.15 (1.02–1.30)	0.02	1.14 (1.01–1.29)	0.04	1.13 (1.00–1.28)	0.05
Hemoglobin, per 10 g/L decrease	1.18 (0.99–1.41)	0.07	1.16 (0.97–1.39)	0.11	1.14 (0.95–1.37)	0.16
Diabetes Mellitus	1.23 (0.78–1.94)	0.38	–	–	1.18 (0.74–1.89)	0.49
Coronary Artery Disease	1.31 (0.82–2.08)	0.26	–	–	1.25 (0.78–2.01)	0.35
Atrial Fibrillation	1.52 (0.96–2.40)	0.07	–	–	1.38 (0.86–2.21)	0.18
Use of RAAS Inhibitors	0.85 (0.51–1.42)	0.54	–	–	0.88 (0.52–1.48)	0.62
Use of Beta-blockers	1.12 (0.68–1.85)	0.65	–	–	1.15 (0.69–1.92)	0.59

The proportional hazards assumption was tested and satisfied for all variables using Schoenfeld residuals. Model 2: Global *χ*^2^ = 45.3, *p* < 0.001; Model 3: Global *χ*² = 52.1, *p* < 0.001 (Likelihood Ratio Test).

* Model 2 adjusted for age, sex, body mass index (BMI), systolic blood pressure (SBP), NYHA Class III/IV, estimated glomerular filtration rate (eGFR), and hemoglobin.

† Model 3 adjusted for all variables in Model 2 plus diabetes mellitus, coronary artery disease, atrial fibrillation, and use of renin-angiotensin-aldosterone system (RAAS) inhibitors and beta-blockers. CI, confidence interval; HR, hazard ratio.

### 3.4 Predictive performance and clinical utility of the combined indicator

The predictive performance of the combined indicator was systematically evaluated and compared against the individual components (E/e' ratio alone and LVMI alone). As shown in Table 3, the combined indicator model demonstrated superior discriminative ability, with a Harrell's C-index of 0.74 (95% CI, 0.68–0.79), which was significantly higher than the C-index of the E/e' alone model (0.67; 95% CI, 0.61–0.73) and the LVMI alone model (0.65; 95% CI, 0.59–0.71). The difference in C-index between the combined model and the best single-parameter model was 0.07 (*p* = 0.008 for comparison with E/e' model), exceeding the pre-specified clinically relevant threshold of 0.05.

**Table 3:** Comparison of predictive performance for HFpEF rehospitalization.

Model	Harrell's C-index (95% CI)	Continuous NRI (95% CI)	IDI (95% CI)
E/e’ Ratio Alone	0.67 (0.61–0.73)	Reference	Reference
LVMI Alone	0.65 (0.59–0.71)	–	–
Combined Indicator	0.74 (0.68–0.79)	0.42 (0.18–0.65)	0.08 (0.04–0.13)
Difference (Combined vs. E/e’)	0.07 (*p* = 0.008)	–	–

The NRI and IDI values are for the comparison of the Combined Indicator model against the E/e’ alone model. CI, confidence interval; IDI, integrated discrimination improvement; NRI, net reclassification improvement.

Furthermore, the combined indicator provided significant improvement in risk reclassification. The continuous NRI was 0.42 (95% CI, 0.18–0.65; *p* < 0.001), indicating a substantial correct reclassification of events and non-events. The IDI was 0.08 (95% CI, 0.04–0.13; *p* < 0.001), confirming that the combined model offered a meaningful increase in the separation of predicted risks between patients who were rehospitalized and those who were not.

**Figure 3:**
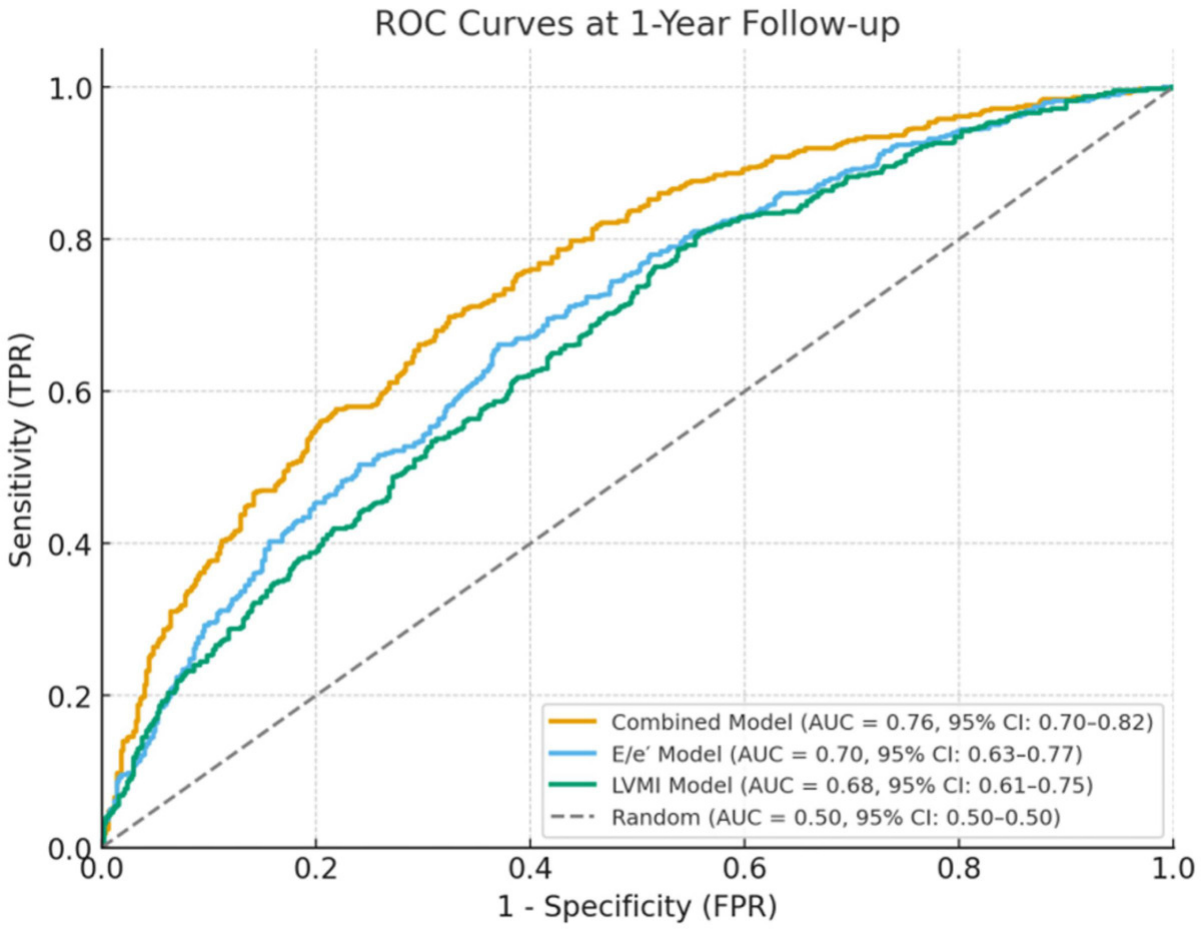
Time-dependent ROC curves at 1-year for HFpEF rehospitalization prediction. The curves depict model discrimination for the primary endpoint, with sensitivity (true positive rate) plotted against 1—specificity (false positive rate). The diagonal dashed line represents the performance of a non-informative random classifier. Solid lines correspond to the combined model, the E/e′ model, and the LVMI model. The legend reports the area under the curve (AUC) with corresponding 95% confidence intervals, as calculated from time-dependent ROC analysis.

The time-dependent ROC curves at 1 year of follow-up are displayed in Figure 3. The combined model achieved the highest AUC of 0.76 (95% CI, 0.70–0.82), compared to an AUC of 0.70 (95% CI, 0.63–0.77) for the E/e' model and 0.68 (95% CI, 0.61–0.75) for the LVMI model, visually reinforcing its superior predictive accuracy for the primary endpoint at this clinically relevant time point.

**Figure 4:**
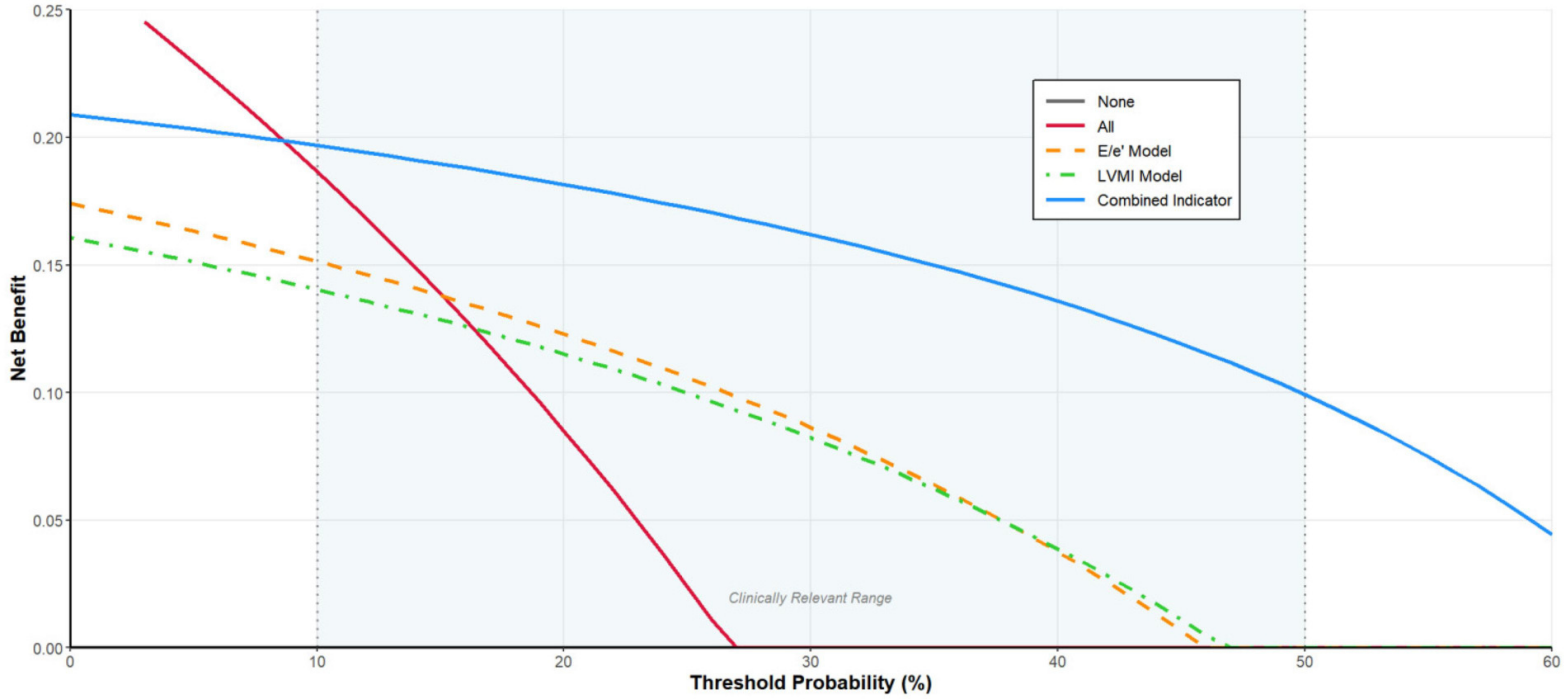
Decision curve analysis for the prediction models. Decision curve analysis showing the net benefit of different prediction strategies across threshold probabilities for HFpEF rehospitalization risk. The *y*-axis represents the net benefit. The *x*-axis represents the threshold probability (%). The gray line (“None”) assumes no patients experience the event. The red line (“All”) assumes all patients experience the event. The blue line represents the combined echocardiographic indicator model, while the orange dashed line and green dot-dash line represent the E/e’ and LVMI single-parameter models, respectively. The light blue shaded area highlights the clinically relevant threshold probability range (10%–50%). Vertical dotted lines mark the boundaries of this range. E/e’, ratio of early transmitral flow velocity to early diastolic mitral annular velocity; HFpEF, heart failure with preserved ejection fraction; LVMI, left ventricular mass index.

Most importantly, DCA was performed to evaluate the clinical net benefit of using the combined indicator for risk stratification. As depicted in Figure 4, the combined indicator model provided a higher net benefit across a wide range of threshold probabilities (approximately 10% to 50%) compared to the strategies of assuming all patients or no patients would be rehospitalized. Notably, the net benefit of the combined model was also superior to that of the single-parameter models over most threshold probabilities, demonstrating its potential clinical utility for guiding decisions on intensified monitoring or preventive strategies in this high-risk population.

### 3.5 Subgroup analysis

**Figure 5:**
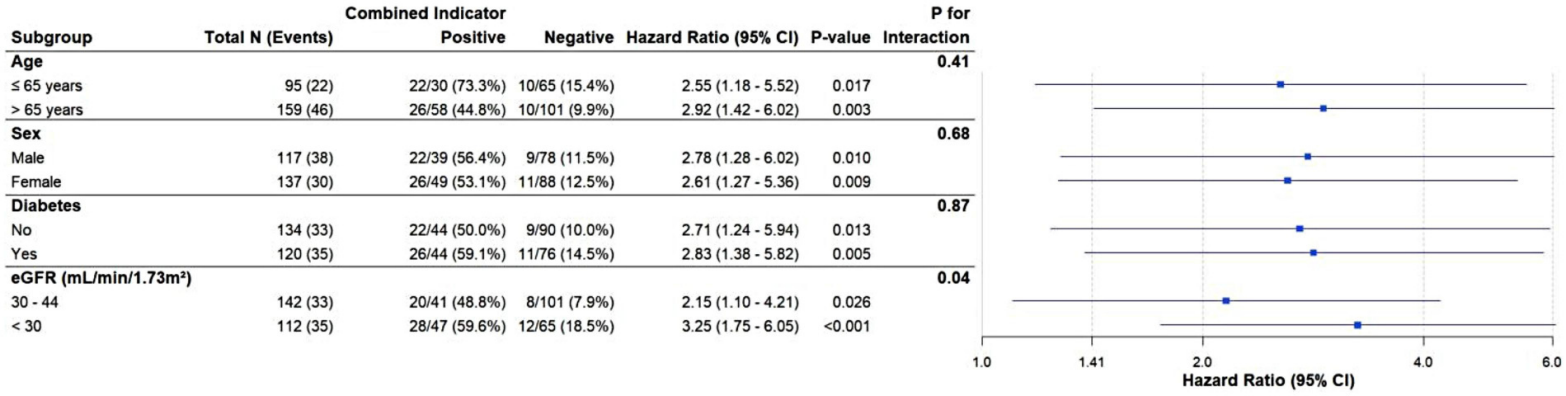
Subgroup analysis of combined indicator on rehospitalization risk. The forest plot displays hazard ratios (HR) and 95% confidence intervals (CI) for the association between the combined indicator and HFpEF rehospitalization risk across predefined subgroups. Each square represents the point estimate of the hazard ratio for the respective subgroup, with the size of the square proportional to the statistical weight of the estimate. Horizontal lines extending from each square represent the 95% confidence intervals. The vertical dashed line at HR = 1.0 indicates no effect (null hypothesis). Hazard ratios greater than 1.0 (to the right of the vertical line) indicate increased risk of rehospitalization, while ratios less than 1.0 indicate decreased risk. The table on the left presents detailed information for each subgroup including sample sizes with event counts, proportions of events in combined indicator positive and negative groups, hazard ratios with 95% confidence intervals, individual *p*-values, and *p*-values for interaction testing. Hazard ratios and 95% confidence intervals were estimated using Cox proportional hazards models adjusted for all variables in the fully adjusted model (Model 3) except for the stratification variable itself. *P*-values for interaction were calculated by introducing a cross-product term (combined indicator × subgroup variable) into the full Cox regression model to test for effect modification across subgroups. Statistical significance was defined as *p* < 0.05. CI, confidence interval; eGFR, estimated glomerular filtration rate; HFpEF, heart failure with preserved ejection fraction; HR, hazard ratio.

Subgroup analyses were performed to assess the consistency of the association between the combined echocardiographic indicator and the risk of HFpEF rehospitalization across various clinically relevant patient strata. As presented in Figure 5, the positive combined indicator was consistently associated with an increased risk of rehospitalization in most predefined subgroups, with HRs significantly greater than 1. A significant interaction was observed for CKD severity (P for interaction = 0.04). The association was notably stronger in patients with more advanced renal dysfunction (eGFR <30 mL/min/1.73 m^2^; HR, 3.25; 95% CI, 1.75–6.05) compared to those with moderate CKD (eGFR 30–44 mL/min/1.73 m^2^; HR, 2.15; 95% CI, 1.10–4.21). In contrast, no significant interactions were detected for age, sex, or diabetes status (all P for interaction > 0.05), indicating a consistent prognostic effect across these subgroups. The point estimates for the HRs in all subgroups remained above 2.0, supporting the robust association between the combined indicator and poor outcomes.

### 3.6 Sensitivity analyses

A series of sensitivity analyses were conducted to assess the robustness of the primary findings. As summarized in Supplementary Table S1, the association between the positive combined echocardiographic indicator and the risk of HFpEF rehospitalization remained statistically significant and materially unchanged across all scenarios. When accounting for the competing risk of death using a Fine-Gray subdistribution hazards model, the fully adjusted subdistribution hazard ratio (sHR) was 2.58 (95% CI: 1.54–4.32). To address potential bias from informative censoring due to loss to follow-up, multiple imputation under the missing-at-random assumption yielded a hazard ratio (HR) of 2.61 (95% CI: 1.57–4.35), which was nearly identical to the primary complete-case analysis result (HR 2.65). Finally, when the HFpEF cohort was redefined using the updated 2021 ESC/HFA diagnostic criteria, the combined indicator remained a strong independent predictor, with a fully adjusted HR of 2.71 (95% CI: 1.62–4.53). These sensitivity analyses confirm the consistent and robust predictive value of the combined E/e' ratio and LVMI indicator.

## 4 Discussion

In this single-center retrospective cohort of patients with stage 3–4 CKD and HFpEF, we developed and validated a novel, readily available echocardiographic indicator that combines the E/e' ratio, a marker of elevated filling pressure, with the LVMI, a marker of concentric remodeling. Most importantly, we demonstrated that this combined indicator not only independently predicted the risk of HFpEF rehospitalization but also provided superior risk stratification compared to either parameter alone, as evidenced by significant improvements in discrimination and net reclassification. This was underpinned by a significant, moderate correlation between E/e' and LVMI (Spearman's *ρ* = 0.68), reinforcing the pathophysiological link between diastolic dysfunction and structural remodeling. However, as visually evident from the wide dispersion of data points around the regression line, the predictive strength of either E/e' or LVMI as an isolated continuous variable can be uncertain and equivocal at the single-subject level. While statistical significance is robust at the population level, the biological heterogeneity among individuals means that relying on a single parameter's value may be clinically misleading for an individual patient. This precise individual-level uncertainty further strengthens the rationale for our combined binary approach. While the prognostic value of both the E/e' ratio and indices of left ventricular hypertrophy (such as LVMI) are well-documented individually in HFpEF (18, 19), most previous studies have examined them in isolation. Our study advances this field by demonstrating that the integration of a functional marker (E/e') and a structural marker (LVMI) captures a broader spectrum of the disease pathophysiology. This synergistic effect likely explains why our combined indicator outperformed each component alone, suggesting that a multi-parametric approach is necessary for optimal risk assessment in this complex population (20).

The pathophysiological rationale for combining E/e' and LVMI is robust, particularly in the context of CKD. LVMI reflects the cumulative burden of pressure and volume overload, which drives concentric hypertrophy and myocardial fibrosis, both of which are central to the structural remodeling in HFpEF (21). This structural alteration leads to increased myocardial stiffness and impaired relaxation, the direct functional consequences of which are captured by an elevated E/e' ratio, signifying increased left ventricular filling pressures (22). In patients with CKD, this adverse cycle is amplified by non-hemodynamic factors such as systemic inflammation, oxidative stress, and uremic toxins, which further promote both myocardial fibrosis and diastolic dysfunction (23). Therefore, the combination of LVMI and E/e' provides a comprehensive assessment by capturing both the cause (structural remodeling) and the hemodynamic consequence (functional impairment) of the disease process, offering a more complete picture of the underlying pathology than either marker alone. By integrating these functional and structural thresholds into a single binary parameter, we provide a streamlined and pragmatic tool for risk stratification. This combined approach offers a clear, actionable clinical indicator that simplifies the decision-making process, allowing clinicians to bypass the complexity of interpreting independent, and sometimes discordant, continuous variables in the complex setting of advanced CKD. The robust association persisted after rigorous adjustment for key prognostic factors in the fully adjusted Cox model (HR 2.65), and notably, our multivariable analysis also confirmed the independent prognostic value of higher NYHA class and lower eGFR, aligning with established literature and underscoring the multifactorial nature of HFpEF rehospitalization risk. This finding resonates with data from large-scale cohorts such as the CORALYS registry, which similarly identified chronic kidney disease and atrial fibrillation as powerful, independent predictors of heart failure hospitalization even in patients with acute coronary syndromes (24). The consistency of these risk factors across different cardiovascular phenotypes underscores the universal prognostic impact of the cardiorenal axis. This integrated approach aligns with the growing consensus that multi-parametric models are essential for unraveling the heterogeneity of HFpEF and improving risk stratification (25).

We acknowledge that the absolute increase in the C-index (0.07) and AUC is modest, and the 95% confidence intervals between the combined and single-parameter models overlap, although increments >0.05 have been suggested to hold clinical relevance in certain prediction contexts (26). The relevance of this increment is particularly pronounced in patients with advanced CKD, a population in which traditional biomarkers like NT-proBNP are frequently confounded by reduced renal clearance, leading to non-specific elevations that do not necessarily reflect acute volume overload (27). Consequently, reliance solely on natriuretic peptides or standard HFpEF scores (which heavily weight such biomarkers) may result in suboptimal risk stratification in this specific cohort. By incorporating specific echocardiographic markers of structural and functional remodeling, our combined indicator provides necessary specificity that complements the limitations of biochemical markers in the cardiorenal setting.

Beyond traditional measures of association and discrimination, our study provides critical evidence for the clinical utility of the combined indicator. The significant net reclassification improvement (NRI = 0.42) and integrated discrimination improvement (IDI = 0.08) confirm that the model meaningfully improves the accuracy of individual risk categorization. Most compellingly, decision curve analysis demonstrated a clear net benefit across a clinically relevant range of threshold probabilities (10%–50%). However, given the modest increment in discrimination and overlapping confidence intervals, its strength in independent clinical decision-making should not be overstated. Taking into account these statistical considerations and the multiple acknowledged limitations of our study, the combined indicator is most appropriately considered as a clinical flag for planning a more intense treatment strategy and follow-up program, rather than a standalone deterministic tool. Furthermore, the consistency of the association across most prespecified subgroups, as illustrated by the forest plot, enhances the generalizability of our findings. The significant interaction observed with CKD severity is particularly intriguing; the stronger association in patients with eGFR <30 mL/min/1.73 m^2^ suggests that the cardiotoxic effects of advanced CKD may amplify the deleterious interplay between myocardial structure and function, making our combined indicator especially relevant in this highest-risk subset.

Our findings must be interpreted within the context of several limitations. First, this is a single-center, retrospective study, and as such, is susceptible to selection bias and unmeasured confounding. Notably, patients with a positive combined indicator exhibited a significantly worse baseline profile, characterized by older age, lower eGFR, and higher prevalence of atrial fibrillation. Although our multivariable models adjusted for these specific covariates, we acknowledge that the combined indicator may, in part, reflect a proxy for overall disease severity and cumulative burden rather than a solely isolated pathophysiological signal. Thus, despite rigorous statistical adjustment, the potential for residual confounding cannot be entirely dismissed. Furthermore, we acknowledge that defining HFpEF rehospitalization in multimorbid CKD patients can be challenging due to overlapping symptoms. To mitigate the potential subjectivity of this endpoint and rigorously handle competing non-cardiac causes of hospitalization (e.g., pneumonia or acute kidney injury), we utilized a strict manual adjudication process. Two independent physicians verified that heart failure was the primary driver of admission based on objective evidence (such as the need for intravenous diuretics), ensuring that hospitalizations driven primarily by non-cardiac etiologies were excluded from the primary outcome. However, the robustness of our findings was unequivocally supported by a comprehensive set of sensitivity analyses. The consistent results from the competing risk model, multiple imputation for missing data, and the redefinition of HFpEF using contemporary criteria significantly mitigate concerns regarding the influence of competing events, informative censoring, and diagnostic criteria, thereby strengthening the validity of our primary conclusions (28). Second, the generalizability of our findings warrants caution. As this study was conducted in a single Chinese center with specific referral patterns, the results may not fully extrapolate to Western HFpEF populations due to potential ethnic differences in body composition (e.g., BMI thresholds for LVH) and healthcare system characteristics. Furthermore, given our specific focus on moderate-to-severe renal dysfunction (stages 3–4), the predictive utility of this combined indicator in patients with milder CKD (stages 1–2) remains uncertain and requires separate validation. Therefore, external validation in larger, multi-ethnic cohorts is warranted to confirm the broader applicability of our combined indicator (29). Third, despite standardized protocols, echocardiographic measurements are subject to inter- and intra-observer variability. Although we took measures to minimize this, such variability is an inherent limitation of the technique. Fourth, the primary endpoint of rehospitalization is inherently influenced by local hospital admission policies, bed availability, and physician-specific clinical thresholds, which may vary across different healthcare settings and potentially modify the model's predictive capability. Additionally, our retrospective design could not account for the dynamic impact of post-discharge clinical management. As a clinical corollary, if patients identified as high-risk by our combined indicator had received proactive planning, including more intensive outpatient follow-up and timely therapeutic modifications (e.g., dynamic diuretic adjustment), their actual incidence of acute decompensation and subsequent rehospitalization might have been significantly reduced, which would paradoxically alter the observed predictive outcomes. Furthermore, the relatively short median follow-up duration of 2.1 years in our cohort primarily captures short- to medium-term rehospitalization risk but limits our ability to evaluate the long-term predictive value of the combined indicator for late cardiovascular events or survival. Extended follow-up in future prospective studies is necessary to fully establish its long-term prognostic utility. Finally, we must acknowledge the inherent limitation of applying population-level statistical trends to individual clinical risk prediction. As highlighted by the wide dispersion of values in our cohort, the predictive strength of individual echocardiographic parameters remains equivocal for any single subject. Although our combined indicator improves prognostic discrimination, it serves as an adjunctive risk stratification tool rather than a definitive deterministic test for individual clinical outcomes.

Despite these limitations, our study has important clinical implications and opens several avenues for future research. The proposed combined indicator is simple to calculate, uses readily available echocardiographic parameters, and could be easily integrated into routine clinical practice to identify high-risk patients with CKD and HFpEF who may benefit from more intensive monitoring and targeted therapies. Future research should first focus on external validation of this combined indicator in prospective, multi-ethnic cohorts. Subsequently, its utility could be tested in a clinical decision-making framework, for instance, to identify high-risk patients who could be prioritized for closer follow-up or enrollment in trials of novel therapies targeting myocardial hypertrophy or fibrosis. With the advent of effective treatments for HFpEF, such as SGLT2 inhibitors, which have shown benefit across the spectrum of ejection fraction, risk stratification tools like ours will be critical for guiding the application of these therapies to patients who stand to benefit most, thereby advancing the goal of personalized medicine in this challenging patient population (30, 31). In conclusion, our study demonstrates that a combined echocardiographic indicator of E/e' and LVMI provides superior risk stratification for rehospitalization in patients with CKD and HFpEF. This simple, yet powerful, tool holds promise for improving clinical management and guiding future research in this high-risk population.

## 5 Conclusion

This study establishes that a novel combined echocardiographic indicator, integrating the E/e' ratio and LVMI, serves as an independent predictor of rehospitalization in patients with concomitant stage 3–4 CKD and HFpEF. The significant correlation between these two parameters underscores the pathophysiological link between diastolic dysfunction and structural remodeling in this high-risk population. The strength of this indicator lies in its significant association with outcomes and its modest but statistically significant incremental performance compared to either parameter alone, as demonstrated by enhanced discrimination, significant net reclassification improvement, and a demonstrable net benefit across clinically relevant decision thresholds. The prognostic value of the combined indicator was consistent across most patient subgroups, particularly pronounced in those with advanced renal impairment, and robust to a range of sensitivity analyses.

This readily available tool provides a holistic assessment of the key pathological processes in HFpEF and offers a simple, practical means for improved risk stratification. Pending validation in prospective cohorts, the integration of this combined echocardiographic indicator into clinical practice could serve as a practical flag for planning a more intense treatment strategy and follow-up program, thereby moving toward a more personalized management approach for this challenging and growing patient population.

## Data Availability

The raw data supporting the conclusions of this article will be made available by the authors, without undue reservation.
