# Supplementary material for: E/e' ratio combined with left ventricular mass index predicts HFpEF rehospitalization risk in stage 3–4 chronic kidney disease patients: a retrospective cohort study
Source: Front Cardiovasc Med. 2026 May 28;13:1758192. doi: 10.3389/fcvm.2026.1758192 (PMC13253623; doi:10.3389/fcvm.2026.1758192)
Supplement: Supplementary file 1 [file Table1.docx]

Supplementary Material

**Supplementary Table 1.** Sensitivity analyses of the association between the combined echocardiographic indicator and HFpEF rehospitalization risk

| **Sensitivity Analysis Scenario** | **Model** | **Hazard Ratio**  **(HR) or Subdistribution HR (sHR) (95% CI)** | **P-value** |
| --- | --- | --- | --- |
| Primary Complete-Case Analysis | Model 3† | 2.65 (1.59 – 4.41) | <0.001 |
| Competing Risk Analysis (Death as competing event) | Model 1 (sHR) | 3.48 (2.16 – 5.61) | <0.001 |
|  | Model 2* (sHR) | 2.85 (1.74 – 4.68) | <0.001 |
|  | Model 3† (sHR) | 2.58 (1.54 – 4.32) | <0.001 |
| Multiple Imputation for Loss to Follow-up | Model 1 (HR) | 3.51 (2.18 – 5.66) | <0.001 |
|  | Model 2* (HR) | 2.88 (1.75 – 4.73) | <0.001 |
|  | Model 3† (HR) | 2.61 (1.57 – 4.35) | <0.001 |
| Redefinition of HFpEF per 2021 ESC/HFA Criteria | Model 1 (HR) | 3.55 (2.20 – 5.73) | <0.001 |
|  | Model 2* (HR) | 2.93 (1.78 – 4.82) | <0.001 |
|  | Model 3† (HR) | 2.71 (1.62 – 4.53) | <0.001 |

Note: The primary complete-case analysis result (from Table 2, Model 3) is included for comparison. The competing risk analysis used a Fine-Gray subdistribution hazards model. Multiple imputation was performed under the missing-at-random assumption using 20 imputed datasets. The cohort redefined by the 2021 ESC/HFA criteria included 248 patients. *Model 2: Adjusted for age, sex, body mass index, systolic blood pressure, NYHA Class III/IV, eGFR, and hemoglobin. †Model 3: Adjusted for all variables in Model 2 plus diabetes mellitus, coronary artery disease, atrial fibrillation, and use of RAAS inhibitors and beta-blockers. sHR, subdistribution hazard ratio; CI, confidence interval.
